# Dr. Chisholm on the Climate and Diseases of Tropical Countries

**Published:** 1822-06-01

**Authors:** 


*A Manual of the Climate and Diseases of Tropical Countries; in which a Practical View of the Statistical Pathology, and of the History and Treatment of the Diseases of those Countries, is attempted to be given; calculated chiefly as a Guide to the Young Medical Practitioner on his first resorting to those Countries.* By Colin Chisholm, M.D. F.R.S. Honorary Member de Physique et d'Histoire Naturelle de Geneva; Member of the Helvetic Society for the Promotion of Science of Switzerland; of the Philosophical, Medical, and Natural Societies of New

York and Philadelphia; and late Inspector-General of Ordnance Hospitals in the West Indies, &c. &c. &c. One closely printed Volume, octavo, pp. 336. London, 1822.

(First Analytical Article.)

With a rooted attachment to his native soil, Man has yet an insatiable avidity for exploring distant regions. Such is the strange compound of his nature! Placed here without armour or defence, the most helpless of animals, his intellect has enabled him to ascend to the highest regions of the air, to sail on the billows of the ocean, to descend into the bowels of the earth. With comparatively an infant's strength, he harpoons the whale, tames the elephant, imprisons the tiger. The feeble hand of man imitates the great operations of nature—he blows up rocks, levels mountains, turns the bottom of the sea into dry land, and leads the waving sail of commerce through the very heart of the country, without the aid of bay, lake, or river!

But it must be confessed that, with man's physical weakness, there is blended a considerable pliancy of constitution, which enables him, by the aid of his reason, to bend to the circumstances around him, and thereby render them less injurious to mind and body. He cannot become a cosmopolite by the mere plastic nature of his frame, without the assistance of his genius. "L'Homme doit à la flexibilité de sa constitution l'avantage d'être cosmopolite, ou de pouvoir vivre dans toutes les regions du globe; mais il ne jouit aussi de cette prérogative sur tous les animaux, que parce qu'il sait se defendre des influences les plus rigoureuses des climats, par des vêtemens, des habitations, par le feu qui le rechauffe et cuit ses alimens, par les soins, la culture, les defrichemens qui assainissent des terrains inhabitable."*

It will not be disputed that the medical officers of the army and navy have contributed much to our knowledge of medical topography, and of the influence of climate on the health and life of our species. Dr. Chisholm, in particular, has long been known as an able, zealous, and intelligent inquirer and observer of all things connected with our Western Colonies; and in committing to the press this result of his experience and reflections (now that he has retired at an advanced age from the fatigues of practice,) he has laid the rising generation of the profession under a debt of gratitude to his memory, which will long continue to be acknowledged

* Virey.

and discharged, when, alas! the corporeal ear will be equally deaf to praise or censure!

The work before us is dedicated by permission to His Royal Highness the Duke of York, and prefaced by a letter to his long tried friend, Sir James Macgrigor, at whose suggestion this Manual has been published. Dr. Chisholm confines himself to the *Western* hemisphere, partly because he never visited the *East*, but principally because he considers the *latter* subject as, in a manner, exhausted by a modern publication well known to the profession. In this work our author informs us that nothing has been advanced, the truth of which has not been known to himself, or to others on whom he could rely. He has, however, strengthened his own propositions occasionally by quotations from, or references to, many of our best modern authors.

Did our Journal circulate only within the limits of the British Isles, the subject matter of Dr. Chisholm's work would still furnish an extensive article of great interest to the home practitioners. But when it is considered that there is not an island in the great West Indian Archipelago which this Review does not visit, it becomes doubly necessary that we should dedicate a space to the volume before us commensurate with its value and importance to all classes of our subscribers.

Dr. Chisholm's work is divided into two great divisions or parts, the *first* embracing the statistical pathology, and the *second* the diseases of tropical countries, more especially the West Indies. These divisions are branched out into chapters and sections on the particular subjects, of which there is a great variety—more indeed than we shall be able to notice even in a cursory manner here.

The West India Islands, Dr. C. observes, may be classed into the high and low, in respect to surface; and into the argillaceous and coralline in respect to structure. The conical summits of the mountains denote, of course, a volcanic origin, and exercise a powerful influence on the salubrity of the atmosphere. Some of these islands, as St. Lucia, Grandeterre of Guadaloupe, portions of Martinique, Dominica, Tobago, Trinidad, and Grenada, are more remarkable than others for marshes, and consequently for insalubrity.

"But the great agent of insalubrity in the higher islands, is the irregularity of their temperature. The windings of the innumerable hills, produce a change of temperature, as they recede into hollows, or project into prominences, giving a quick and most unpleasant alternation, of almost unsupportable heat, and consequent profuse perspiration, and comparative cold with dry and corrugated skin. Another cause of insalubrity in the mountainous islands, is the humid state of their atmosphere, occasioned by the attraction of their peaks or conical summits and clifts, and extensive forests." P. 2.

Antigua being altogether argillaceous, is distinguished by a peculiarly cold atmosphere, comparatively speaking, producing on the human constitution all the effects of marsh miasma, although no swamps exist in the neighbourhood of St. John's, where this singular cold is chiefly felt after rain. The view of the country of Lamantin from the height of Morne Garnier, in the Island of Martinico, before the sun has penetrated the fog, excites astonishment how any human being can exist there.

"Nothing is perceived but the summits of the hills, every other object lying hid under a vast expanse of dense white fleecy damp vapour:—if a calm prevails, the fleecy atmosphere is immoveable; but if the gentlest breeze springs up, an undulation takes place, and presently huge volumes accumulate, and slowly roll along, carrying their pestiferous miasms towards Fort Royal, or mingling them in the waters of the Bay. The consequence of this is, the temperature of Fort Royal, and the immediately adjacent country, is subject to great variation; and the chilliness or aguish cold which prevails here during the night, excites the most unpleasant sensations imaginable." P. 3.

The temperature of the West India Islands is regulated, of course, by the degree of elevation. The medium heat on the coast is 84°; while at the accessible parts of the higher mountains, it is about 60°—a range of 24 degrees. This is an important fact when we come to consider the preparation of the unassimilated European to the Torrid Zone.

"I shall here further observe, that this gradation of temperature, according to the altitude of surface, is principally efficient in rendering the West-India islands very healthy, when no foreign cause of disease exists, and when the inhabitants are exposed to that only which is endemic. That they would prove so to every description of their inhabitants, European and native, British and French, were they all equally careful to avoid those excesses, and those imprudences in diet and personal exposure, which, in all climates, are dangerous, and too often fatal, may be demonstrated in many instances. The uninterrupted health and longevity of the French and Creole inhabitants of both sexes, are the result of an active, prudent, and temperate life. Eighty, ninety, a hundred years, is by no means an uncommon age among these people—nor does this appear to be owing to their residence being higher and cooler, than that of the British, although that, as a general cause of health, is, as I have already remarked, particularly remarkable; for many European French, and some Creoles, possessing fine plantations, on the coast, enjoy the same exemption from disease." 3.

The North-East, or *windward* shores of the West India islands are more level or declivous than the leeward or S.W. sides—they are consequently more marshy and unhealthy. Exposed to the northerly winds of spring, their inhabitants are annually, at that season, afflicted with pulmonic and hepatic inflammations. The dry season, in the West Indies, is from the beginning of December till the end of April; and is usually pleasant and healthy. The approach of the rainy season, in the two last months of summer, is awful, and always indicated by thick fogs hanging about the summits of the higher mountains, followed by dark, watery clouds, rolling from the North-East in terrific volumes, and darting bright electric corruscations from their edges. Tremendous torrents of water are suddenly precipitated from these clouds, and, rolling down the bottom of ravines and beds of rivers, carry every thing before them, discolouring the sea for several miles in every direction. The wet season, in the West as in the East, is checkered by many successive days of insupportable closeness and sultriness. The months of March and September are particularly stormy in all the islands, and the tract from 11° South to 18° North, is too often visited by those tremendous hurricanes which carry terror and devastation in their train by land and sea. The medium height of the thermometer at one P. M. in the shade is 83° varying from 74 to 92.

The second chapter of Part I. is on the tendency of tropical climates to the production of disease. Dr. Chisholm remarks that, if we abstract the miasmata of marshes and the abrupt vicissitudes of temperature, the *first* giving rise to endemic fevers, and the *second* to local inflammations, the mortality is very little more, in ordinary years, than in England. This position presumes, and indeed Dr. Chisholm directly expresses it, that solar heat, except under particular circumstances, "has never been a cause of disease." But in the subsequent page our ingenious author acknowledges that the same solar heat powerfully *predisposes* the European constitution to be acted on by other causes of disease, and we would ask in what does this *predisposition* consist? Is it not evidently a deterioration of the constitution—in other words, a morbid condition, or, in fact, a *disease* of the animal frame?* Every man, indeed, who has accurately remarked

* Our author states that the action of solar heat *alone* does directly produce what has been termed *coup de soleil;* (carus ab insolatione) and if so, we ask if it be not probable that a long-continued application of the same agent, though in a degree too small to produce coup de soleil, may yet produce other changes of a morbid nature in the system, corresponding with this milder degree of the solar agency?

his own feelings, or consulted the feelings of others, under high atmospheric temperature between the tropics or any where else, must be convinced that such feelings are incompatible with long and regular health. We ask all those who have sojourned between the tropics whether there is not something of a wearing, wasting, and exhausting nature in high atmospheric temperature, which not only predisposes to the action of other morbific causes, but actually conducts to premature old age and decay, should the individual be fortunate enough to escape the more obvious and unequivocal diseases of tropical climates induced by other agents than high temperature. With this qualification we agree with our author that, speaking generally, atmospheric heat only lays the *predisposition*, while terrestrial exhalations and vicissitudes of temperature call into *action* the principal diseases of tropical climates.

There is another draw-back on Dr. Chisholm's comparative salubrity of the West Indies—namely, his omission of those "malignant pestilential fevers," which, from time to time, have ravaged the said islands, and which he attributes to imported contagion, but which many others attribute to the climate itself. If *these* be included in the effects of climate, then the comparative salubrity of inter-tropical and northern countries drawn by Dr. Chisholm will not hold good. We will not, however, dip into this long litigated question, but proceed to the *third* chapter, which takes up the subject of *Hygiene*.

Dr. Chisholm's observations correspond with those of the best observers, that those men are most subject to the higher grades of the remittent as well as the pestilential fevers of the Western hemisphere, who are unhabituated to the Torrid Zone, and who possess plethoric habits, sanguineous temperaments, and rigid fibres. The grand principle of our author's Hygiene will be gathered from the following short extract.

> "I have said that solar heat, although seldom a direct, should always be deemed an indirect morbid cause, in as much as it powerfully predisposes the system of the native of a cold or temperate climate to be acted on by the direct endemic or imported causes of disease, he may be exposed to on his arrival in the tropical. It follows, therefore, that the lessening the action of heat on the system of such a person, constitutes the principle on which assimilation depends; and in its consequence gives a ready solution to all the phenomena of seasoning." 10.

This is the very principle laid down by Dr. James Johnson also, in his work on tropical climates. "The two fundamental rules of tropical hygiene, says Dr. J. are *tempe-*

*rance* and *coolness*—the latter indeed includes the former, and simple as it may appear, it is the grand principle of inter-tropical hygiene. From *heat* spring all those effects which originally *predispose* to the operation of other morbific causes. And how can we obviate these effects of *heat*, but by calling in the aid of its antagonist, *cold?*" It is always satisfactory to see two authors, both independent and original in their observations, agree on material points, whether of pathology, therapeutics, or hygiene.

Our author very properly remarks, that tranquillity of mind and body, for some time after arriving in a tropical climate, is a necessary condition to give effect to a plan of preparation founded on the above principle—hence the difficulty of effecting this object among troops employed immediately after their arrival, on actual service:—"during which, the excessive heat and fatigue in the day, and the cold and humid evaporation in the night, to which they are alternately exposed, together with the large quantity of animal food which enters into their diet, give a manifold increased aptitude to suffer by endemic or imported causes of disease." Our author thinks, and with great probability, that could the exigencies of the state permit the indulgence of a few month's ease and tranquillity, with a cooling, refreshing diet, to troops on their arrival from Europe, in some island, as for instance, Barbadoes, not particularly subject to irregular temperature, a great deal might be done to prevent the danger in assimilating their systems to the climate. We copy the following prophylactic measure from our able author. The plan has been ridiculed by some writers, whose lucubrations, however, are fast sinking into oblivion, if, indeed, they are now remembered at all.

"On reaching the northern tropic of N. lat. 23°, every stranger to the torrid zone should be bled to an extent proportioned to his age and strength; and a pill of five grains of calomel, given at night, and a saline purgative the following morning. The bleeding should be repeated, if necessary, once before landing; but the calomel and salts should be frequently resorted to; and this will be more necessary should there be a disposition to constipation. I have already observed that on approaching the tropics, a considerable tendency to congestion is perceived:—this greatly increases on a further advance, more especially hepatic congestion, which, in fact, is the most serious consequence to be apprehended on entering the tropics.—Nothing more effectually obviates this, than moderate bleeding, and mercurial and saline purgatives. To assist this course, the diet should be made as cooling as possible. Perspiration being the great means employed by nature to carry off the superfluous heat, every thing which tends to restrain it should be avoided; dilution is, therefore, in every respect, highly necessary; and it is evident, that, with this view, water is the fluid best calculated, for whilst it promotes perspiration, it necessarily prevents determinations and congestions." 12.

Cold bathing is recommended, and properly, we think, by Dr. C. as an essential item of prophylaxis. After landing, it is desirable, if possible, to place the men out of the reach of marsh effluvia, and so high as to secure a temperature of from 70° to 80°. Exercise should there be frequent and regular, with *gradual* exposure to the sun. We recommend this part of the work to commanding officers of regiments or corps, as containing most sound, rational, and practical doctrines.

The medical management after landing, must be analogous to that during the voyage. Plethora must be diminished by occasional bleeding and purging—the cutaneous surface kept clean and permeable by bathing, and by the use of flannel and oily inunctions.

The fourth chapter is on the particular seasons of the West Indies. From accurate returns and eighteen years' personal observations, it appears that the months of July, August, and September, are the most sickly—Oct. Nov. and Dec. next in degree; and Jan. Feb. and March least sickly. In the next page, by some error of the press, it is recommended to send troops *before* the month of October, and a little farther on, the best time of leaving England is stated to be early in October. It is probable that a *not* is omitted in one of these passages, which, of course, alters the sense of the text entirely.

The endemic diseases of the West Indies are either bilious or inflammatory as the seasons are hot and wet, or cool and dry. Thus in the summer and autumn we have remittents, dysenteries, cholera, &c. and in marshy districts, at this season, obstinate intermittents almost always depending on visceral obstruction or inflammation, together with hepatic dysenteries of a very dangerous character. In the winter and spring the complaints are, of course, of an inflammatory character.

Miasmal diseases are by far the most formidable within the Torrid Zone. The yellow and sallow complexions of those who may be considered as "ascripti glebæ," manifest the nature of the air they breathe; and the short lives, of the men more especially, constitute a still more forcible testimony. Unfortunately those bays and inlets which afford most protection from the stormy elements to our shipping, are those which are most productive of dreadful miasmal diseases. The carenage or harbour of Fort Royal, in Martinique, exhibits a melancholy illustration of this truth.

A stranger, Dr. C. justly observes, who believes that the diseases of tropical climates must necessarily spring entirely from heat, will be greatly disappointed. In fact, the best writers on those climates represent a great proportion of the diseases of Europeans as proceeding from the application of *cold*, after the human frame has been enervated by heat.

"3. The third sub-division, viz. the diseases from cold and moisture within the tropics, also demand a no less assiduous attention. Exposure to this state of the atmosphere has always been found injurious to the human constitution in hot climates. In these climates, this combination has never failed to produce the effects of marsh influence, united with the inflammatory diathesis proceeding from cold alone. In fact, its product is typhoid inflammation, which is always topical, and often attended by symptomatic fever. The form the inflammation assumes is either pleurisy, hepatitis, or rheumatism, and the type of fever is remittent generally, often intermittent. The intestinal canal is not unfrequently the seat of this inflammation, and almost always in the form of dysentery, so often experienced by armies obliged to lie on wet ground, and after exposure to rain and great fatigue." 22.

Imprudence and intemperance are prolific sources of disease within as well as without the tropics. Exposure to a very high temperature during the day, and chilling dews in the night—especially if combined with intemperance in drink, is too often followed by dangerous yellow fever, or no less fatal hepatitis, terminating, with rapidity, in gangrene of the liver. Dysentery and enteritis are also very frequently the consequence, and often end quickly in mortification of the bowels. For many excellent prophylactic remarks at the conclusion of this chapter, we must refer to the work itself. The fifth chapter also we must pass over, (embracing the prevention of disease depending on public policy,) particularly as it takes up the subject of *contagion*, on which we shall not at all enter in this article—for unfortunately the more the subject has been discussed, the greater has been the discrepancy of opinion entertained, and the more tenacious have the opposite parties become of the doctrines originally embraced. We do not therefore deem it adviseable to waste our time in hopeless disputations.

The second part of Dr. Chisholm's work is on the diseases of the West Indies, and especially those of an endemic nature. Those, he observes, might be divided into four classes as before—namely, as resulting from marsh exhalation, atmospheric vicissitudes, cold and moisture united, and lastly, intemperance, &c. but, in truth, the causes of disease are so often mingled within the tropics, that the subdivision in question is not always so distinct as to render it necessary to treat of the endemic diseases accordingly.

I. *Yellow Remittent Fever.* This naturally occupies the first rank. The etiology, symptomatology, pathology, and treatment, are ably pourtrayed by our most intelligent author, and will form an admirable manual to our younger brethren on first entering the Western Tropics. It is hardly necessary to say, that the grand agent in the production of this fever, in all parts of the world, is that invisible, inexplicable something, designated " vegeto-animal, or marsh effluvium," which issues from the soil of tropical climates, and even temperate climates, at certain seasons when atmospheric temperature rises towards the tropical range.

As to the symptomatology of these fevers, it is unfortunately too well known to the British profession to need any recapitulation here. The pathology has appeared to our author very uniform, from the inspection of a great number of bodies.

" The principal organ affected was the liver: and it is not a little singular, that in those cases of the worst kind of this fever, which terminated fatally, this viscus was found either in a loose, dissolved putrid state; or sphacelated, and having the consistence, the feel, and colour of rotten cork! or full of abscesses:—in such cases, too, the biliary ducts were rendered impervious by stricture; little bile in the gall-bladder, and that always black, ropy, and granulated. In many cases, several portions of the intestinal canal were inflamed, particularly the duodenum; and here and there evident marks of gangrene were observed. The spleen was greatly enlarged. The mesenteric glands were, generally, enlarged, in a state of scirrhus, or full of pus. The stomach, in general, had its coats thickened, the villous coat abraded, and the blood vessels much distended. No bile was found in it;—but black mucus, or the fluid discharged resembling coffee grounds. Every part of the body appeared tinged with a deep yellow colour.—It was remarkable that the blood, in the large vessels, was in very small quantity, and had more the appearance of serum or water, tinged with a yellowish red colour, than of blood." 44.

In many of these fevers, whether endemic or epidemic, other authors have found the brain bearing the marks of the vascular excitement which occurred in the course of the fever.

*Treatment.* The first object, Dr. Chisholm justly observes, is to freely evacuate the primæ viæ, and deplete the vascular system. If the patient be seen early, " a copious bleeding and plentiful evacuation by stool, very often put a stop at once to the disease." " If, however, the disease persists, the bleeding must be repeated, and alvine evacuations again plentifully procured."

"When, at this period, irritability of the stomach seems likely to prevent the retention of the purgative medicine, it should be always preceded by about a grain of opium, or a draught containing 20 or 30 drops of laudanum. If the disease still appears disinclined to yield, the mercurial plan must be adopted without delay,—but further bleeding is generally unnecessary or hurtful—five grains of calomel, with or without opium, according to the state of the stomach and bowels, are then to be given in a little treacle or syrup, and repeated every two, three, or four hours, according to the urgency of the symptoms, and the degree of danger apprehended.—Thirty or forty grains have, generally, brought on ptyalism.—When this happens, all the alarming symptoms disappear. In three or four days after, the patient becomes convalescent—but the disease has too often proved obstinate under this milder treatment.—When, therefore, the symptoms of a more formidable fever appear, and the danger is evidently imminent, the dose of calomel should be increased even to 20 or 30 grains every third or fourth hour; and, if the vomiting increases, various means should be employed to allay the irritation of the stomach. These means are opium, ether, effervescing draughts, blisters to the inside of the thighs, and the very frequent use of common laxative clysters, or of those consisting of a watery solution of assafœtida.—But mercury, assisted by cold affusion, must be mainly trusted to; and its exhibition, under the untoward circumstance of an irritable stomach, must be varied in every possible way—by injection, by friction, without measuring or attending to the division, of the strongest ointment, into portions, but rubbing it on every part of the body, ad libitum, until the effect, on which alone safety depends, is produced. I can confidently assure the young practitioner, that not a single patient in my practice, died, even under the worst form of the disease, if mercury could be introduced in sufficient quantity to produce ptyalism. But the practitioner must not be afraid to use this medicine with the utmost freedom in such cases;—he must have confidence in it, and persevere until the object is obtained. Abundant fœculent discharges are necessary, whilst mercury is producing its specific effect; and this may be done by mercury (calomel) alone; or, if there is constipation, by the addition of jalap, or the extract of colocynth: but an opposite state of the bowels, is, by far, more frequent, and rather requires the restraining power of opium.—I may sum up the treatment, I have uniformly adopted since the year 1791, in Yellow Remittent Fever, thus: it is my first intention to relieve the system in general, by plentiful and reiterated bleeding and purging; but having effected this, during the first 24 or 30 hours of the disease, long experience has convinced me, that it is upon a new action being excited that the safety of the patient depends. Although many instances have occurred, of a copious and protracted diaphoresis, or an abundant and sudden flow of urine, having removed every complaint, yet I was chiefly, perhaps always, directed in forming a favourable prognosis, by the supervention of mercurial action on the gums and salivary glands.—Many instances have occurred to me, which have taught me not to despair whilst the most distant hopes remained of accomplishing that: and, therefore, where this action has been tardy, and where there are, at the same time, symptoms of the most imminent danger, I have endeavoured to introduce the medicine in every possible way, and assisting the means I employed by cold bathing, and, if necessary, by the use of spiced wine and nourishing food made as acceptable to the stomach as possible." 46.

Our own observations confirm the truth of the above therapeutics, which we recommend to the serious attention of the young tropical practitioner.

The second chapter of this division of the work is on intermittents, and contains many excellent remarks and judicious instructions. When the fever intermitted regularly, whatever was the type, our author exhibited the cinchona or arsenical solution (the latter is the more powerful of the two) after proper evacuations. When the paroxysms did not cede to this, a pill composed of colocynth extract and pil. hyd. was ordered every night at bed time—a precaution very necessary, Dr. C. observes, when the bark is given.

" But when the succession of paroxysms is very frequent, the intermissions very imperfect, and no indisposition appears in the fever to become otherwise, local congestion and inflammation should be considered as the real disease, and the irregular fever as purely symptomatic. In this case, no time should be lost in giving mercury, with such freedom, and so guarded with opium, if necessary, as to excite ptyalism, as soon as possible. This medicine, thus given, has never failed to stop the progress of the disease. It has, unfortunately, happened, sometimes, that the disease has been so insidious, that the second pasoxysm, after an imperfect intermission, has completely overwhelmed the patient, and put an end to his existence.—I have therefore made it a rule of practice, in these irregular fevers with aggravated symptoms, to ascertain with anxious minuteness, whether pain and fulness exist in the hepatic region—and if they do, to begin the treatment with copious bleeding and purging; and then to proceed to the use of mercury, in the same confident manner as in the yellow remittent fever.—The disease, in truth, arises from the same cause, is then marked with the same symptoms, produces the same morbid changes, is possessed of the same danger, and consequently must be treated in the same manner." 51.

In our author's experience, there were few or no relapses when the cure was effected by mercurial ptyalism after proper evacuations ;—owing, he conceives, to the removal of local disease, of which the more violent kinds of intermittents, as well as remittents, are symptomatic. Removal, however, from the sphere of the miasmal exhalation is necessary to prevent relapses in all cases. The usual sequelæ of fever, as visceral obstructions, bowel complaints, &c. do not occur when the fever has been checked by a mercurial action in the system. Dr. Chisholm has seen the most unequivocal proofs of sol-lunar influence on intermittents within the Tropics.

*Chap.* III. *Dysentery.* Dr. Chisholm observes that there are two species of dysentery between the tropics—one proceeding from suppressed perspiration, irregularities in diet, clothing, &c. exposure to currents of air when the body has been heated. The other species, and by far the more dangerous, occurs in the hot and rainy season, and has for its cause, Dr. Chisholm thinks, the miasmata of marshes. The first is sporadic—" and the proximate or immediate cause seems evidently to be irritation of the larger intestines from an overcharge of their vessels, and consequent inflammation of their coats." " The second is always a symptomatic disease, having its seat in the liver and small intestines." Although we believe the *function* of the liver to be implicated in all cases of dysentery, we cannot bring ourselves to go the length of Dr. Chisholm's position that this organ is the exclusive seat of the disease in those epidemic dysenteries which spread devastation through marshy countries.

Dr. Chisholm's description of the idiopathic dysentery (as distinguished from the hepatic or miasmal) does not differ from that of other writers; and the same may be said of the *post-mortem* appearances. The following extract will exhibit one mode of treating the mild species, where circumstances render the mercurial treatment inadmissible.

" Although the early symptoms of dysentery are generally deceitful, and although, therefore, it is the part of prudence to employ a remedy of well ascertained efficacy in the treatment; yet I shall here detail the means that may be resorted to in cases evidently of a mild nature, or in which the proximate cause may be supposed or deemed, superficial inflammation of the lining membrane of the intestinal canal, when mercurial ptyalism is inadmissible, from constitutional latent disease, or from the prejudice or fears of the patient. No disease manifests the sympathy between the skin and the intestinal canal, more than dysentery. The great object, therefore, should be to restore the skin to a soft permeable state; for in this disease it is generally, indeed I may say, always, dry and corrugated. When a gentle, warm diaphoresis is excited, the patient always experiences relief; the motions become less mucous, and more fœculent. When this method of cure is resolved upon, it may be conducted in the following manner.—After the full operation of an emetic, and a purgative, five grains of James's powder may be given every four or five hours, till a copious, warm diaphoresis is thrown out, which ought to be kept up by plentiful dilution with warm rice or barley water, for several hours, or until the griping and tenesmus cease, and the motions assume a better, that is, a less mucous and a more fœculent appearance.—This state being produced, the bark may be given in pretty large doses, mixed with port wine or with water; and a pill, such as the following, thrice in the day, or only once at bed time, according to the circumstances of the case: ℞. opii gr. ss. ad. gr. 1 pulv. Jacob. gr. iij. balsam Peruv. q. s. f. pilula. When the sweat is slow in breaking out, it may be promoted by hot fomentations;—or what is better, by wrapping the belly and lower extremities in a blanket, wrung out of boiling water, or a hot decoction of aromatic herbs.—The efficacy of the last application in relieving the symptoms, and bringing on a diaphoresis, I have often witnessed in a very remarkable degree. An excellent mode of exciting diaphoresis, without the disagreeable inconvenience of having the body enveloped in wet, is by means of the vapour bath, which may be very conveniently and agreeably employed in the manner described in the chapter on rheumatism." 57.

Another mode of treatment in recent cases, is an emetic of ipecacuanha or sulphate of zinc, given in the evening, after which, from four to ten grains of the Dover's powder may be administered at bed-time, with from two, to six or seven grains of calomel mixed together in a spoonful of treacle. Our author recommends small doses of sulphate of magnesia the next day, if some stools are not procured by the calomel —after which, the Dover's powder and calomel are to be again repeated in the evening. For many other therapeutical measures we refer to the volume itself.

*Hepatic Dysentery.* A fixed pain at the pit of the stomach and constant head-ache, at the very commencement of the disease, are the only symptoms which serve to distinguish hepatic from idiopathic dysentery, according to Dr. Chisholm.

" In other respects, it does not seem to differ from the idiopathic or common dysentery; so that it can almost never be known but by the experienced, until those symptoms appear, which, whilst they manifest the peculiar nature of the disease, also, unhappily, point out its approaching fatal termination. It is, therefore, I repeat it, of the highest importance, when the two symptoms I have mentioned appear at the commencement of dysentery, to ascertain the local peculiarities of the patient's place of residence—that is, whether the patient has resided, and contracted the disease in the immediate vicinity of marshes, and what are the diseases which have most frequently occured in the situation—whether there is any epidemic at the time. If the situation is marshy, and the epidemics have been, or are, remittent and intermittent fevers, and hepatic complaints;—then, the pain at the pit of the stomach and headache, accompanied by a disposition to frequent alvine dejection, should be considered as indicating hepatic dysentery. Every chance of success depends on the early detection of the disease, and, of course, the early adoption of the treatment which experience has proved to be the only useful one. Towards the fatal period, which, in the worst form of hepatic dysentery, comes on with an overwhelming rapidity, the sudden and unexpected supervention of a general coldness of the surface, with partial cold clammy sweats, an almost total cessation of pulse, an excessive sinking of the spirits, and a discharge from the bowels, composed of a slimy brown substance, floating in a fluid like bloody water, and having a fetor of intolerable offensiveness, mark the irremediable progress the disease has made. This is the worst state of the disease, in which no human means can avail—a less unfavourable form of the disease presents some symptoms less equivocal, and which more early discovers its nature. The commencement, and for three or four days, the symptoms are those usually observed in dysentery; but at the end of that time, together with the pain at the pit of the stomach and the headache, a considerable anxiety at the præcordia, and a sensation as of a continued pressure in the right hypochondrium, with frequent stools, composed of a fluid like the washings of raw meat, are perceived. These symptoms should be particularly noticed, and deemed as truly characteristic of the seat of the disease being the liver. It will be happy for the patient, and creditable to the practitioner, if they are so; and if they are made the regulating principle of the treatment. But if these signs are neglected, and the fatal ones permitted to come on, nothing can be useful; and the progress of the disease from thence to death is most rapid, indeed, so much so, that from the appearance of these signs, death takes place in six, ten, or at the most twenty-four hours."

On dissection, our author found the "liver of all viscera the most diseased," being inflamed, enlarged, partially suppurated, or, in some portions, sphacelated. The whole intestinal canal was more or less inflamed, especially the small intestines.

"The mode of treatment I adopted in this obscure disease, after I became acquainted with its nature, was the following. After bleeding once or twice, if the pulse indicated the repetition, a vomit of tartarized antimony, or of what I found generally preferable, sulphate of zinc, and a sufficient purge of castor oil, the following pills were given.—℞ submur. hydrarg. gr. iij. pulv. ipecac. gr. iv. opii, gr. ss. mucilag. q. s. ft. pilulæ duæ. The two were given every three hours during the twenty-four. Emollient glysters, such as the following were administered thrice in the day—℞ amyl. solut. ℥vi. tinct. opii, gt. lx. ad c. pulv. ipecac. gr. viii. ℳ ft. enema. If the danger was imminent, and the symptoms increased in urgency, the dose of calomel was augmented in such manner as to excite ptyalism as quickly as possible. When this took place, danger ceased, and the patient soon became convalescent. I first began to use this mode of treatment in the year 1786, and have most successfully continued it ever since. It is now more generally known, and every judicious practitioner employs it." 60.

The pathology of dysentery "drawn up by a learned and ingenious friend of the medical staff of the army," is almost verbatim and literatim that which was given by Dr. Johnson seven years ago, in his work on tropical climates, and we wonder that Dr. Chisholm, whose candour and impartiality are every where conspicuous, should have, in this instance, failed to observe the good old rule—" *suum cuique*."

*Hepatitis*. The next subject discussed by our very intelligent author is hepatitis. We are much inclined to coincide, though not to the full extent, with Dr. Chisholm in the following dogma, sweeping as it may appear to those who have not well considered the subject. "All the endemic diseases of hot climates have their immediate origin in the over-excitement of the hepatic and cuticular systems—fever, dysentery, and hepatitis, are thus produced." He properly observes that the whole of the abdominal viscera are so connected in their functions, that if an important organ is excited to unhealthy action, all the rest sympathize with it, and the usual consequences, inflammation, congestion, or morbid secretion, follow.

"Taking a more extended view of the pathology of the abdominal viscera, we find, that an intimate connexion subsists between the organic system of the skin, and that of the liver; insomuch, that whatever disturbs the operations of the former, necessarily and concurrently disturbs those of the latter." 63.

We shall give, in his own words, Dr. Chisholm's rationale of chronic hepatitis in hot climates, which, as most of our readers will readily perceive, coincides very remarkably with that of Dr. Johnson, detailed in the work on tropical climates abovementioned.

"Chronic inflammation of the liver is generally the result of active or acute, and may be thus explained. From previous over-excitement of the hepatic and cuticular systems, torpor and atony, the invariable consequences follow—the secretion of healthy bile is suspended, and the expulsion of that already secreted becomes slow, or altogether ceases. The viscid and tenaceous bile thus retained, stagnates in the biliary vessels, or is taken up by the absorbents into the general circulation, causing the yellowness of the conjunctiva of the eyes, and of the skin; whilst the intestines, deprived of their natural stimulus, become torpid, and accumulation of fæces and flatus follows.—Hypochondriasm, the constant result of such a state of things, discovers its usual phœnomena, mental depression, solicitude about health, visions of evil, apprehension of death, and despairing consciousness and fear of future punishment. It is, indeed, wonderful, in this state of the hepatic system, how readily the mind receives deep and afflictive impressions of religious enthusiasm;—

the most gloomy ideas are cherished, although, perhaps, the conduct of life may have been correct and virtuous. I have frequently witnessed this state, and as frequently dissipated it, by rousing the liver into a due performance of its natural functions. Melancholia religiosa, which, in fact, is only another name for hypochondriasm, occasioned by this morbid state of the liver, should never be considered as a disease of the mind. It is physical not moral derangement;—and, if the proper remedy is so applied as to restore the diseased organ to healthy action, this, the effect of that derangement, will disappear." 64.

Here Dr. Chisholm describes an anomalous hepatitis, or what we should rather call *hepatic fever*, which he has several times seen both epidemically prevailing, and sporadically occurring in the West Indies, especially among blacks, and young people from the age of 8 to 25—a peculiarity, doubtless, owing to these people being more exposed to the causes, which were observed to be exposure to cold and marsh miasmata.

"The disease began with a considerable degree of head-ache, pain at the pit of the stomach, and tightness across the præcordia, with difficult respiration. The skin was dry, corrugated and cool; the tongue moist and foul; the belly natural, and the discharge of urine free. No thirst, no sickness, scarcely any diminution of appetite; the pulse soft and not more than 70 or 80, and of a natural fulness. On the second day, the head-ache increased much; the pain at the pit of the stomach became excruciating; cold shivering came on; the skin, on pressure, communicated an intense, penetrating heat, although, on slightly touching, it felt cold, and its surface excessively dry and corrugated; the tongue covered with a thick moist fur, purplish towards the edges, and grey in the middle. In negroes a bronze or coppery colour on the cheeks, out of which large drops of clammy sweat issued, whilst a greasy moisture overspread the rest of the face; in whites, the colour of the face became dingy with the same kind of moisture—the pulse quickened at once from 80 to 120 and 144, and became hard and contracted. A short cough, or rather a sudden, quick respiration came on, with a sensation at the diaphragm, as if a heavy weight pressed on the lungs, and was about to suffocate the patient. On the sixth day, if the patient lived so long, the pulse suddenly sunk, so as to become almost imperceptible; the greasiness of the face increased, a glassiness appeared in the eyes; a disagreeable cold clamminess over the whole surface took place; a great increase of weight at the diaphragm, and a sense of stricture in the pharynx, with excessively difficult deglutition succeeded; and the whole closed with coma and death. The proportion of deaths to recoveries was as one is to six; and the fatal days were the 3rd, 5th, 7th, 11th. Repeated observations and numerous dissections alone directed to a knowledge of the nature of this most treacherous disease.—The morbid appearances were very uniform.—The liver astonishingly enlarged; on its surface, particularly the convex side, an irregular intermixture of red purplish and tallow coloured spots, exhibiting a marbled appearance; yet the texture, although the whole so much enlarged, as, in eight cases out of ten, to occupy both hypochondria and the epigastrium, was otherwise, in a natural state, and without the smallest vestige of suppuration or gangrene. All the other abdominal viscera were in a sound state. The diaphragm seemed, indeed, in most cases inflamed, and its blood-vessels distended with blood.—Every other part of the body had a healthy appearance." P. 66.

After numerous attempts by other means, the following was found to be the best mode of treatment. The patient was bled as soon as possible, until deliquium animi or relief of symptoms was obtained—for which purpose forty or fifty ounces were frequently subtracted at one bleeding. A blister was then applied to the side, and cooling tartarized medicines given, repeating the venesection, pro re nata, so that in the course of three or four days our author has known from 40 oz. to 10℔s. of blood drawn. But it was not found eligible to trust to these remedies alone. On the second day, after the third bleeding, if there were not evident signs of amendment, "which indeed very seldom happened," from two to ten grains of calomel made into pills, with or without opium according to the state of the bowels, were given three times in the day. "This practice generally brought on a copious salivation in two days. When this was effected the patient was safe. It was astonishing how readily cases of the most dangerous tendency yielded to this treatment; and it was no less so, how quickly the sick recovered their usual health and strength, notwithstanding the great loss of blood they sustained; while many who had been bled more sparingly continued in a languid state for months."

"It soon became evident, however, that this remedy alone, as I have already observed, could not effect the cure; for when mercurial ptyalism did not take place, while bleeding was liberally employed, recovery was extreme'y tedious, or the patients died on the 7th or 11th day.—Upon the whole, bleeding to a degree beyond all common bounds, and promoting a copious salivation as speedily as possible, were the only means of cure we placed confidence in." 67.

In respect to acute hepatitis, it is a little curious that, till about the year 1770, it was unknown—that is, undetected. Yet the causes of the disease must have existed, and been in operation. But in those days it was called "bilious fever," and no *post mortem* investigations were resorted to. In fact, when our author settled in the West Indies, "the most marked symptoms of hepatic inflammation were entirely overlooked"—"a putrid diathesis was alone dreaded, and alone provided against by bark, wine, and other stimulating tonics."

"The natural consequence followed. Hepatic gangrene, hepatic abscess, and hepatic diarrhœa, have destroyed thousands; and yet improper treatment of the original disease, had never been considered as the cause." 68.

We recommend the following short symptomatology of acute hepatitis between the tropics, to the attention of the young visitor in the Torrid Zone.

"Acute hepatitis often comes on without any previous warning; and is distinguished by violent pain in the right hypochondrium, a tightness across the abdomen, and a fulness, difficult respiration, and inability to lie down in any posture, the most easy position being a sitting one, with the upper part of the body inclined forwards. There is no heat, no quickness of pulse at first; but excessive and undefineable anxiety and restlessness. In this form death sometimes happens, on the 3rd or 5th day, from gangrene of the liver.—When the disease approaches more slowly, there are, for some days, the usual precursors of disease, followed by the symptoms of remittent fever, and a sense of fulness and obtuse pain in the right side. At this period, bleeding in a moderate degree, and the operation of a brisk purge, relieve the patient so much, as to induce him to neglect his situation. In a few days, the same symptoms appear again with greater violence; but again disappear by some purging and other antiphlogistic means. All this time, however, the disease is gaining ground, but the apparent mildness of the symptoms, and the ease with which they seem to be removed, throw the patient off his guard; and it often happens that medical advice is not deemed necessary until the disease is completely formed, has considerably advanced, and manifests itself by excruciating pain in the right side, attended with twitchings or spasms, and pain in the right shoulder, great anxiety, excessively difficult respiration, and total inability to lie down. In this state, if the most vigorous measures are not adopted without hesitation, and assiduously pursued, gangrene or suppuration in the liver soon follow." 69.

The treatment of the first form must be prompt and decided—bleeding till the pain is relieved—abundant purging—and mercurial ptyalism, in the shortest space of time possible. Blisters are merely auxiliaries.

The second form admits of more time, and a slower administration of the remedies—"but the remedies must be the same." The following observations are equally important and correct. Many a time have we witnessed the truth of them.

"If there is a tendency to diarrhœa before ptyalism is excited, it must be checked by opium. An important fact, in endeavouring to excite mercurial action, should be carefully kept in view.—If the patient is particularly robust, and of a sanguineous constitution, mercurial action cannot be brought on, until the system is reduced to that level, which permits that action to take place—on the other hand, if the system is low, and the natural constitution of the patient feeble, whilst the hepatic inflammation is going on, a coincidence by no means unfrequent, means of raising it to the level, permitting mercurial action, must be employed whilst the administration of mercury is proceeding. In the first case copious and reiterated bleeding—in the second, the cold infusion of cinchona, and simple though nourishing food, are the means I have found most productive of the desired effect.—Sometimes ptyalism comes on rapidly and unexpectedly, and becomes troublesome—*scarcely ever dangerous.* The means of checking it, are the warm bath, opium, occasionally purging, and the use of a gargle, composed of a decoction of dry figs, with nitre, in the proportion of two drachms to a pint—sulphur and the sulphuret of potash have been seldom useful." 69.

*Chronic Hepatitis* is generally the result of the acute form neglected or improperly treated. In the East Indies, however, and in Europe, this form of the disease *generally* creeps on without any acute symptoms having preceded. A common, but by no means pathognomonic symptom, is a sense of oppression, fulness, and bulk in the right hypochondrium, accompanied by an obscure, obtuse pain, only occasionally evident to the feelings of the patient—especially after strong drink, suppressed perspiration, falls or other external injuries, full meals, violent exercise, &c.

" The following circumstances, however, appear more conclusive, viz. pain felt in the right, on lying on the left side; difficult respiration, or a sudden, quick expiration, following an attempt to inspire deeply; and an exacerbation of all the symptoms at a particular time of the day. The third seems peculiar to chronic hepatitis, and therefore more worthy of our attention, when the other less distinguishing symptoms are also present. The exacerbation takes place, generally, about four o'clock in the afternoon of every day, and continues one, two, or more hours. It is marked by aggravation of the other symptoms, and the presence of considerable heat and quickness of pulse, neither of which are at any other time perceived. But difficult respiration, or the quick and sudden expiration following an attempt to inspire deeply, when a fulness and obtuse pain are felt in the right hypochondrium, I consider as the most pathognomonic of all those symptoms ascribed to acute and chronic hepatitis." 71.

Icteritious colour of the skin and eyes is certainly more peculiar to chronic than acute hepatitis. With this colour there is generally torpor of the bowels, and clay-coloured motions.

" In chronic hepatitis, I may further observe, that there is generally a circumscribed solid substance felt, inclining or suspended, as it may be described, from the right to the left side, on attempting to lie on the latter. It is described by the patient as an unconnected mass, which seems to roll from side to side, according to the position he places himself in;—if bending forward, which is, indeed, the easiest posture in this disease, it seems to press against the parietes of the abdomen;—if towards the left, it inclines that way, and gives considerable pain of that kind, which we may conceive a heavy weight suspended from the right side may give, and is well defined by the expression *dragging pain;*—if backwards, it rests on the spine, and occasions instantaneous acute pain, with tendency to syncope." 72.

The nature of this disease renders it an insidious and dangerous complaint; and thousands perish under its influence, who might probably be saved by timely interference. The following is our author's methodus medendi in chronic hepatitis.

"In the treatment of chronic hepatitis, the two principal objects must be, 1st, to remove any inflammation that may be present in the liver; and 2nd, to rouse it to the natural and due performance of its functions.—The first is obtained by moderate, general, or partial bleeding and purging; and the second, by exciting a gentle mercurial ptyalism, and giving tone to the secreting vessels, by the use, at the same time, of mild tonics, combined with the nitric acid, and interposing every second or third day, a plentiful discharge from the bowels. To excite the degree of ptyalism required, if the bowels are torpid, their usual state in the disease, from two to five grains, or even more of calomel, may be given only thrice in the day; or if diarrhœa is brought on, opium may be added. But in this open state of the bowels, it may be better to introduce the mercury by friction. For this purpose, a drachm of the strongest mercurial ointment should be well rubbed in on the inside of the thigh every night, or oftener, according to the excitability of the patient, until a gentle ptyalism is established.—The following draught should also be taken thrice in the day—℞. infus. gentian.—colomb. āā ℥vi, tinct. cinchon. comp. ℨi, acid. nitrici, gtt. viij. ad gtt. xij. If there is evident tumour in the right side, a seton should be introduced over it. When the bowels are torpid, during this course, the following powder and draught should be given every second or third day, or seldomer, according to circumstances: ℞. submur. hydrarg. gr. ij. ad. v. pulv. rhei. gr. x. M. ft. pulvis horâ somni sumendus—℞. infus. sennæ ℥iss. magnes. sulph. ℨi. tinct. sennæ—card. comp. āā ℨi. M. ft. haustus primo mane post pulverem sumendus.—This mode of treatment I have uniformly found successful, when the disease admitted of cure, that is, if instituted before the supervention of abscess. But, indeed, in very many instances, if the tumour is the result of abscess, it gradually disappears, through the increased action of the absorbents, brought about by this course; or, as happened in a few cases, the pus passes off by the common biliary duct into the duodenum, and thence is discharged by stool.—If, however, the tumour increases, and the abscess proceeds to maturity, without the possibility of arresting it by any internal means, and points outwardly, with evident adhesion to the integuments, then an opening must be made, and the matter discharged." 74.

In the eastern hemisphere and in Europe, a slower introduction of the mercury than is above directed, is generally sufficient; but, it is absolutely necessary to keep the mouth sore till the symptoms are entirely dissipated, else the patient is left in a worse state than before. In chronic organic diseases of the liver in this country—which are often of a tuberculous or scrofulous nature, mercury, and indeed all other medicines are of little avail. Gentle aperients—local abstractions of blood—counter-irritation, and taraxacum with the mineral acids, are the principal remedies.

The remainder of the volume before us is chiefly occupied with a class of diseases as common (or more so) in this country as between the Tropics. We shall therefore reserve it for separate consideration in the succeeding number of this Review. Our European readers, we are persuaded, will have been gratified and improved by what we have extracted from the first half of the volume—they will find in our next article matter of high import, which it will behove them to study well. Our tropical brethren, more especially those in, or proceeding to, the West Indies, may rest assured that this is one of the most valuable publications that ever issued from the press on the diseases and medical topography of the western hemisphere. They will not, therefore, fail to place it in their libraries. To the venerable, zealous, and able author himself, we offer the tribute of unfeigned gratitude, respect, and esteem. He has wound up a long and active life by bequeathing to posterity a legacy that will render that life as useful to others hereafter, as it has been honorable to himself in times past. He has had his "youth of labour," and it is but right that he should now have his "age of ease." He spent the hey-day of health amid the burning savannahs of Western India. He has now but—

> "To husband out life's taper at its close,
> "And keep the flame from wasting by repose,"

amid those peaceful valleys and alpine solitudes that formed the cradle of a ROUSSEAU and the retreat of a GIBBON.

